# Genomic Analysis of Broad-Host-Range Enterobacteriophage Av-05

**DOI:** 10.1128/genomeA.00282-15

**Published:** 2015-06-11

**Authors:** Luis Amarillas, Osvaldo López-Cuevas, Josefina León-Félix, Nohelia Castro-del Campo, Charles P. Gerba, Cristóbal Chaidez

**Affiliations:** a Centro de Investigación en Alimentación y Desarrollo, A.C., Culiacán, Sinaloa, Mexico; b Instituto de Investigación Lightbourn, Chihuahua, Mexico; c Department of Soil, Water and Environmental Science, The University of Arizona, Tucson, Arizona, USA

## Abstract

Lytic bacteriophages have reemerged as an alternative for the control of pathogenic bacteria. However, the effective use of phage relies on appropriate genomic characterization. In this study, we report the genome of bacteriophage Av-05 and its sequence analysis, which has strong lytic activity against *Escherichia coli* O157:H7 strains and several *Salmonella* serotypes. The analysis revealed that the phage Av-05 genome consists of 120,938 bp, containing 209 putative open reading frames (ORFs) and 9 tRNAs.

## GENOME ANNOUNCEMENT

The worldwide rise of antibiotic-resistant bacterial strains has created the need for alternative means of controlling pathogenic bacteria. In recent years, there has been an increasing interest in the use of bacteriophages as promising alternative agents for the control of pathogenic bacteria (1, 2). Recently, phage Av-05 was isolated and biologically characterized. This phage presents strong lytic activity against a wide range of *Escherichia coli* O157:H7 strains and several *Salmonella* serotypes (3). In this study, we report the genome of bacteriophage Av-05 and its sequence analysis. Phage Av-05 was obtained from the strain collection of the National Research Laboratory for Food Safety (LANIIA) at the Research Center for Food and Development (CIAD, Mexico). This phage was isolated from poultry feces collected on farms in Sinaloa, Mexico. Av-05 was propagated and purified by the double-layer agar overlay technique using *E. coli* O157:H7 (CECT 4076 strain) as a host, as described previously (4). Phage DNA isolation was performed with the SDS-proteinase K protocol, as described by Sambrook and Russell (5). Genome sequencing was performed using Genome Analyzer IIx (Illumina, Inc.) technology.

A total of 54,583,412 reads were generated, with a read length of 36 bases, resulting in ~100× coverage of the genome. The generated reads were assembled *de novo* using Geneious R7 (Biomatters Ltd., New Zealand) (6), resulting in a single contig. Potential open reading frames (ORFs) were identified using the server from GeneMark (http://exon.gatech.edu/). The putative functions of the ORFs were compared in the nonredundant sequence database (National Center for Biotechnology Information [NCBI]) to find sequence homology using BLAST, (see http://www.ncbi.nlm.nih.gov/). The presence of tRNAs in the genome sequence was determined using tRNAscan-SE (7) and ARAGORN (8). Additionally, the genome ends were determined as described by Casjens and Gilcrease (9).

The genome of phage Av-05 consists of 120,938 bp, with a G+C content of 40.0%. A total of 209 putative ORFs and 9 tRNAs were found in the phage genome, with a total of 88.92% nucleotides involved in coding for putative proteins (coding density), reflecting the compactness of phage genome organization, as this feature is common to all the phages (10). The Av-05 genome has modular organization, which is common among the phages (11), apparently organized into five major clusters: structural and morphogenesis, DNA packaging, DNA replication, DNA modification, and host lysis. Genome sequence analysis and annotation of Av-05 indicated that 16 of the ORFs identified are involved in viral morphogenesis, with 19 proteins involved in the DNA metabolism, three encoding proteins associated with the DNA packaging, and five probably involved in host lysis. One hundred forty-four of the ORFs were defined as encoding conserved hypothetical proteins, none of which has an identifiable function. The remainder of the ORFs showed no significant homology to other proteins in GenBank. The ORFs revealed a close relationship to proteins of bacteriophage PVP-SE1, indicating that phage Av-05 may be a member of “*Pvplikevirus,*” a new genus related to bacteriophage T4 (12, 13). In comparative analyses of the genome sequence with the phage genomes in the NCBI database, the DNA sequence of the Av-05 genome showed the highest similarity (97% identity) with the RB69 phage. However, the genome comparison between these two phages revealed regions that are different, with the most pronounced difference being in the tail fiber proteins. These differences likely confer upon bacteriophage Av-05 the capacity to have a broad lytic spectrum.

### Nucleotide sequence accession number.

The genome sequence of phage Av-05 has been deposited in the GenBank database under the accession no. KM190144.
